# Construction and Properties of Wood-Based Tannin–Iron-Complexed Photothermal Material *Populus tomentosa Carr*.@Fe-GA for Solar Seawater Desalination System

**DOI:** 10.3390/ma18020393

**Published:** 2025-01-16

**Authors:** Hongyan Zhu, Xinyu Li, Shijie Li, Ximing Wang, Yabo Ma, Jin Zhang, Yunpeng Ren, Jianguo Zhao

**Affiliations:** 1 Inner Mongolia Key Laboratory of Sandy Shrubs Fibrosis and Energy Development and Utilization, College of Material Science and Art Design, Inner Mongolia Agricultural University, Hohhot 010018, China; zhuhy1314211@163.com; 2 Engineering Research Center of Coal-Based Ecological Carbon Sequestration Technology of the Ministry of Education, Shanxi Datong University, Datong 037009, China; lixinyurd@163.com (X.L.); li841974@sina.com (S.L.); ma17851960242@163.com (Y.M.); zhangjing8014484@163.com (J.Z.); ryp1197805948@163.com (Y.R.); 3 Special Graphite Application Technology Innovation Center of Shanxi Province, Shanxi Datong University, Datong 037009, China

**Keywords:** seawater desalination, utilization of wood resources, solar energy, photothermal material, efficient evaporation

## Abstract

Desalinating seawater is a crucial method for addressing the shortage of freshwater resources. High-efficiency, low-cost, and environmentally friendly desalination technologies are key issues that urgently need to be addressed. This work used *Populus tomentosa Carr.* as a matrix material and prepared *Populus tomentosa Carr.*@Fe-GA through a complexation reaction to enhance the water evaporation rate and photothermal conversion efficiency of seawater desalination. The concentration of the impregnation solution was further refined, and the bonding mechanism along with the thermal stability of the composite photothermal material was investigated, including an assessment of their photothermal conversion efficiency. The research results indicate that the evaporation rate of water in a 3.5% NaCl solution for *Populus tomentosa Carr.*@Fe-GA under light intensity conditions of one sun reached 1.72 kg·m^−2^·h^−1^, which was an increase of 44.5% compared to untreated *Populus tomentosa Carr.* It achieved a photothermal conversion efficiency of 95.1%, an improvement of 53.6% over untreated *Populus tomentosa Carr.*, and maintained stability and high evaporation performance (95.4%) even after prolonged rinsing. This work realizes the functional utilization of seawater desalination with *Populus tomentosa Carr.* and offers a novel approach for the development and use of wood-derived photothermal material.

## 1. Introduction

The world is currently facing multiple challenges such as rapid population growth, water pollution, and climate change, which severely limit access to safe drinking water. The limited availability of freshwater resources has emerged as a significant global issue [1,2,3,4,5,6,7,8]. Since the 20th century, seawater desalination technology has gradually developed as a key means to address freshwater shortages, attracting widespread attention and research [9,10,11,12,13,14,15]. However, traditional desalination technologies (distillation, reverse osmosis) have high energy consumption, complex equipment, and high costs, exacerbating energy crises and environmental pollution, thus limiting their feasibility for large-scale applications [16,17,18]. Replacing fossil energy with natural light through photothermal conversion for seawater desalination represents a zero-carbon emission technology, which has led to a growing interest in finding environmentally friendly, economical, and efficient seawater desalination materials [19,20,21,22].

Photothermal conversion technology has garnered significant attention for its ability to utilize solar energy directly for water evaporation [23,24,25,26,27]. Among this, photothermal black material serves as a crucial component of photothermal conversion technology, exhibiting high light absorption, low reflectivity, and excellent thermal conductivity; this enables the transformation of solar energy into heat, resulting in effective energy conversion [28,29,30,31,32,33]. Therefore, applying photothermal blackening material in seawater desalination technology is expected to enhance desalination efficiency and reduce energy consumption and costs [34]. Various substrate materials have been identified, including polymer sponges [35,36], self-assembled aerogels [37,38,39,40], plasma metal decorative films [41,42], and natural derivatives [43,44,45]; this helps water evaporate by encouraging water to enter the pores of the substrate through capillary action at the interface between air and water. Nevertheless, as water accumulates within the matrix, inefficient evaporation and significant thermal losses may occur, leading to a marked decrease in photothermal evaporation efficiency and limiting its application potential [46]. *Populus tomentosa Carr.*, as a natural renewable material, exhibits excellent water absorption and permeability. Its natural microporous structure and fibrous organization can absorb and store a large amount of water, releasing moisture through natural permeation, allowing *Populus tomentosa* wood to regenerate in real time during seawater desalination, thereby reducing the need for manual intervention. This makes it an ideal moisture transport medium and an efficient wood-based photothermal seawater desalination material [47,48]. Studies indicate that combining photothermal conversion technology with renewable materials can significantly reduce energy consumption, demonstrating promising application prospects, especially in real seawater environments.

This work is aimed at developing an innovative and efficient seawater desalination material. By designing a simple and environmentally friendly method to prepare *Populus tomentosa Carr.*@Fe-GA, the limitations of traditional seawater desalination materials and technologies are overcome. The novelty of this work lies not only in the unique combination of a tannin–iron complex with a *Populus tomentosa* matrix but also in the capillary effect of wood treated with NaOH. The synergistic effect between the two enables *Populus tomentosa Carr.*@Fe-GA to achieve an excellent evaporation rate and photothermal conversion efficiency. This work not only provides a promising solution to the global freshwater shortage but also paves the way for the further development of photothermal conversion and renewable energy technologies. The future will focus on optimizing materials and processes to unlock their full potential in various applications related to water treatment, thereby contributing to a more sustainable future.

## 2. Materials and Methods

### 2.1. Materials

Tannic acid (TA, 99%) was supplied by Aladdin (Shanghai, China), and ferric chloride (FeCl_3_·6H_2_O, ≥99%), sodium hydroxide (NaOH, ≥96%), and sodium chloride (NaCl, ≥99.5%) were supplied by McLean Co., Ltd. (Shanghai, China).

The unique pore network structure of *Populus tomentosa Carr.* is conducive to the diffusion and migration of water molecules inside the wood, which effectively improves the water evaporation rate of the material. It was harvested from Yunzhou District, Datong City, Shanxi Province. The local planting area was wide and easy to obtain. The wood sample was taken from 1.5 m above the ground, specifically from the outer part of the xylem. The dimensions of the wood chips were 50 mm in length, 50 mm in width, and 2 mm in thickness.

### 2.2. Preparation of Populus tomentosa Carr.@Fe-GA

**Figure 1:**
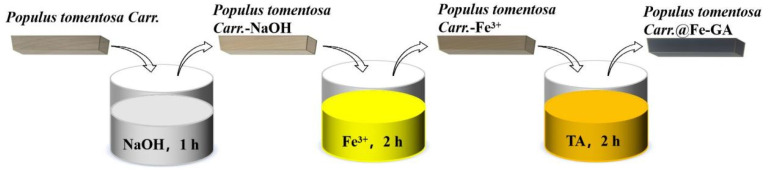
Schematic illustration of preparation process for *Populus tomentosa Carr.*@Fe-GA. (*Populus tomentosa Carr.* is abbreviated to *P*).

*Populus tomentosa Carr.* (dimensions 50 mm × 50 mm × 2 mm) was soaked in a 10% NaOH solution for 1 h, effectively removing lignin and some hemicellulose, thereby exposing more hydrophilic cellulose groups and enhancing the material’s water absorption [49]. After washing with distilled water until neutral, the wood was dried in a vacuum oven at a temperature of 60 °C. To further improve material photothermal properties, the dried *Populus tomentosa Carr.* was impregnated in various concentrations of an FeCl_3_ aqueous solution (0%, 2%, 4%, 6%, 8%) for 1 h and subsequently dried in a vacuum oven at a temperature of 60 °C. It was then immersed in various concentrations of a TA aqueous solution (0%, 2%, 4%, 6%, 8%) for 1 h. The resulting blackened wood chips were rinsed with deionized water until the solution was clear, yielding *Populus tomentosa Carr.*@Fe-GA. The samples were labeled as *Populus tomentosa Carr.*@Fe-GA-X (where X represented the different impregnation concentrations × 100). Figure 1 illustrates the preparation of the *Populus tomentosa Carr.*@Fe-GA photothermal material. The reaction followed the chemical reaction in Equation (1).

C_76_H_52_O_46_ + FeCl_3_ = C_76_H_49_O_46_Fe + 3HCl (1)

### 2.3. Characterization Methods

The surface structure of *Populus tomentosa Carr.* was examined through scanning electron microscopy (SEM, TESCAN MALA 3 LMH, Brno, Czech Republic); the sample was sprayed with gold using ion sputtering apparatus. An energy-dispersive spectrometer (EDS, Oxford INCAx act, High Wycombe, UK) was utilized to examine the chemical makeup of *Populus tomentosa Carr.*, and the measurement parameters were as follows: acceleration voltage, 20 kV; objective distance, 6 mm. Fourier transform infrared spectroscopy (FTIR, Tianjin Port East Company FTIR-650, Tianjin, China) was used to analyze the infrared absorption spectrum of *Populus tomentosa Carr.*, and the measurement parameters were as follows: the scanning range was 4000–400 cm^−1^ and the scanning resolution was 4 cm^−1^. A thermogravimetric analyzer (TG-DSC, STA449, NETZSCH, Selb, Germany) was used to analyze the thermal stability and enthalpy of the evaporation of *Populus tomentosa Carr.*, and the measurement parameters were as follows: test atmosphere, nitrogen; scanning speed, 10 °C/min; test temperature range, 30–800 °C. A thermal infrared imager was used to capture the surface temperature and infrared images (Infrared thermal imaging, FLIR-E6390 from Friel Corporation, New York, NY, USA), with an emissivity of 0.6. UH4150 (UV-DRS, Hitachi UH4150, Tokyo, Japan) was used to analyze the absorption/reflection spectra of *Populus tomantosa Carr.*, and the measurement parameters were as follows: slit width, 2 nm; scanning point interval, 5 nm; scanning range, 200–2500 nm. We used a contact angle to test the hydrophilicity of *Populus tomentosa Carr.* Using a xenon light source system (CHP-XM550, Beijing Changtuo Technology Co., Ltd., Beijing, China) to measure the moisture changes in simulated seawater desalination processes, the light intensity was 1 kW·m^−2^.

### 2.4. Simulation Experiment of Seawater Desalination

**Figure 2:**
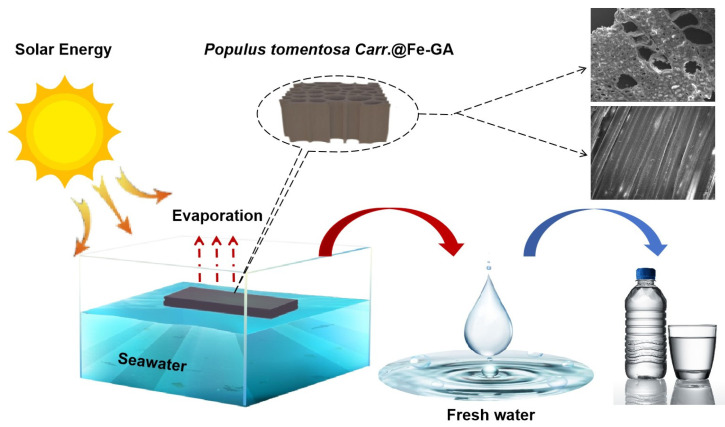
Schematic diagram of *Populus tomentosa Carr.*@Fe-GA evaporation system. (*Populus tomentosa Carr.* is abbreviated to *P*).

This work used cross-sectioned *Populus tomantosa Carr.* as the substrate material, utilizing the wood’s natural hydrophilic moisture transport channels for natural moisture transport, and set up simulated seawater desalination apparatus. A schematic diagram of the *Populus tomentosa Carr.*@Fe-GA evaporation system is shown in Figure 2.

The experiments on seawater evaporation were carried out at a temperature of 25 °C, with a humidity level of 60%, and under a solar intensity of 1 kW·m^−2^ (as recorded by a light flux intensity meter, FGH-1). In the experiments, a 3.5% sodium chloride solution was utilized to mimic seawater, which differed from common pure water experiments and aimed to simulate the photothermal performance of materials under actual seawater desalination conditions. *Populus tomentosa Carr.* was fixed at the bottom of the device (48 mm (length) × 48 mm (width)) to ensure adequate contact with the water surface, and the device was immersed in simulated seawater containing 3.5% NaCl. An electronic scale was utilized to measure the loss of mass throughout the experiment. The evaporation efficiency was calculated using the formula below:

η = m h_lv_/C_opt_ P_0_ (2)

In this context, m represents the mass flux (m = m_light_ − m_dark_) measured in kg·m^−2^·h^−1^, h_lv_ indicates the total enthalpy change during the evaporation process from liquid to vapor, P_0_ refers to the solar irradiance equivalent to one sun, and C_opt_ denotes the optical concentration on the surface of the absorber. To determine the evaporation efficiency of photothermal water, it is essential to subtract the average evaporation rate of pure water measured in a dark environment [50,51]. Furthermore, the evaporation rate (V_e_) is influenced by the mass change in water due to evaporation (Δm), the area of the photothermal material (S), and the duration of evaporation (t).

V_e_ = Δm/S × t (3)

## 3. Results and Discussion

### 3.1. Study on Evaporation Performance of Populus tomentosa Carr.@Fe-GA

**Figure 3:**
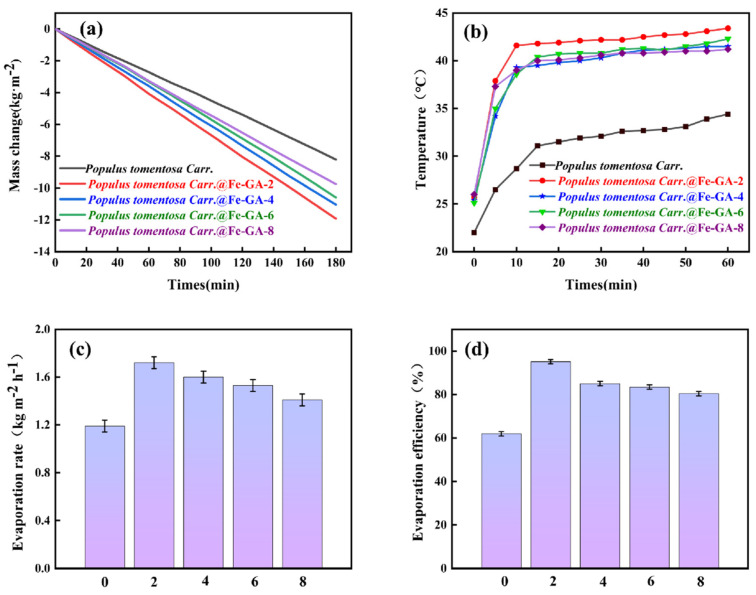
*Populus tomentosa Carr.* and *Populus tomentosa Carr.*@Fe-GA-(2,4,6,8). (**a**) Water mass loss map. (**b**) Temperature rise curve. (**c**) Water evaporation rate curve. (**d**) Photothermal conversion efficiency. (Solar power density measured is established as one sun). (*Populus tomentosa Carr.* is abbreviated to *P*).

To explore the photothermal performance of *Populus tomentosa Carr.*@Fe-GA, we constructed a simulated seawater desalination device. With irradiation levels below 1 kW·m^−2^, water could rapidly ascend from the lower part to the upper photothermal layer of the photothermal material via the vertical channels present in the wood., relying on capillary action to replenish the evaporation loss at the top. Figure 3a shows the changes in mass loss for *Populus tomentosa Carr.* and *Populus tomentosa Carr.*@Fe-GA-(2, 4, 6, 8). The recorded evaporation rates of water in direct sunlight (with a 3.5% NaCl solution) were 1.19, 1.72, 1.60, 1.53, and 1.41 kg·m^−2^·h^−1^ (as shown in Figure 3c), all of which were considerably greater than the evaporation rate of simulated seawater, which was 0.27 kg·m^−2^·h^−1^. Among them, the evaporation rate of *Populus tomentosa Carr.*@Fe-GA-2 was the best, showing an improvement of 44.5% compared to untreated Populus wood. According to Equation (2), *Populus tomentosa Carr.*@Fe-GA-2 attained a photothermal conversion efficiency of 95.1% at a light power density of 1 kW·m^−2^. To calculate this efficiency, the water evaporation rate of the device in the absence of light (0.30 kg·m^−2^·h^−1^) was deducted to account for the effects of natural water evaporation, which was an increase of 53.6% compared to untreated Populus wood, indicating that this photothermal material can fully utilize solar energy.

The temperature rise curve (Figure 3b) also confirms the above results; the temperature rise rate of *Populus tomentosa Carr.*@Fe-GA-2 was faster than that of *Populus tomentosa Carr.* and *Populus tomentosa Carr.*@Fe-GA-(4, 6, 8), and the surface temperature of *Populus tomentosa Carr.*@Fe-GA-2 could reach up to 43.4 °C after 60 min, indicating its excellent moisture evaporation performance. Additionally, the outstanding photothermal conversion efficiency of *Populus tomentosa Carr.*@Fe-GA is attributed to several factors: (1) The tannin–iron complex created during the reaction had a strong ability to absorb solar light and quickly transformed solar energy into thermal energy. (2) The vertically arranged channel structure within the wood effectively reduced the resistance to water transport. (3) The insulating properties of the wood minimized heat loss to the water body. This work will focus on *Populus tomentosa Carr.*@Fe-GA-2, which is assumed to be *Populus tomentosa Carr.*@Fe-GA.

### 3.2. Bonding Mechanism Between Tannin–Iron and Populus tomentosa Carr.

**Figure 4:**
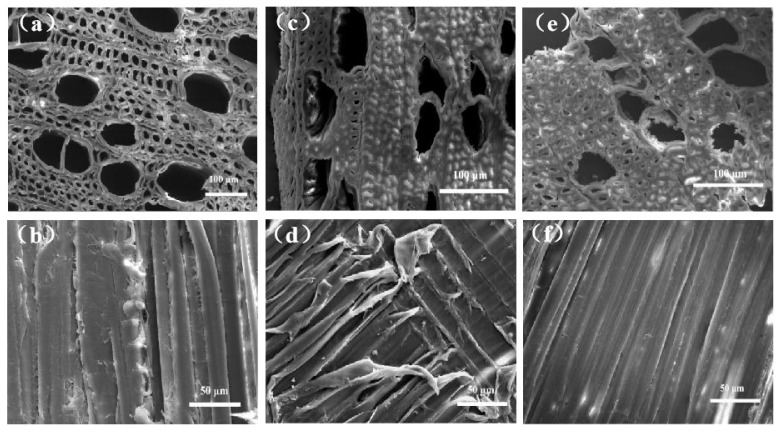
SEM images of (**a**,**b**) *Populus tomentosa Carr.*; (**c**,**d**) *P*@NaOH; and (**e**,**f**) *P*@Fe-GA (*Populus tomentosa Carr.* is abbreviated to *P*).

Figure 4a,b are scanning electron microscope images of transverse and longitudinal sections of *Populus tomentosa Carr.*, revealing the distribution of moisture transport conduits and capillary channels in the wood. Figure 4c,d are SEM images of *Populus tomentosa Carr.*@NaOH, demonstrating that the removal of lignin and some hemicellulose led to a lightweight laminated structure in the wood, and the pore diameter of the wood significantly decreased after NaOH treatment. SEM images of *Populus tomentosa Carr.*@Fe-GA are shown in Figure 4e,f, where substances are evenly loaded in the original moisture transport conduits of the wood. The presence of Fe elements in *Populus tomentosa Carr.*@Fe-GA (Table 1) could be attributed to the high quantity of hydroxyl groups present on the wood’s surface, which facilitated the accumulation of TA, leading to the formation of complexes with Fe^3+^. The reduction in pore diameter enhanced capillary action, facilitating the absorption and transport of water molecules. The formed tannin–iron complex was loaded onto the inner walls of the pores, successfully improving the wood’s ability to absorb light, which in turn boosted the moisture transport rate.

**Figure 5:**
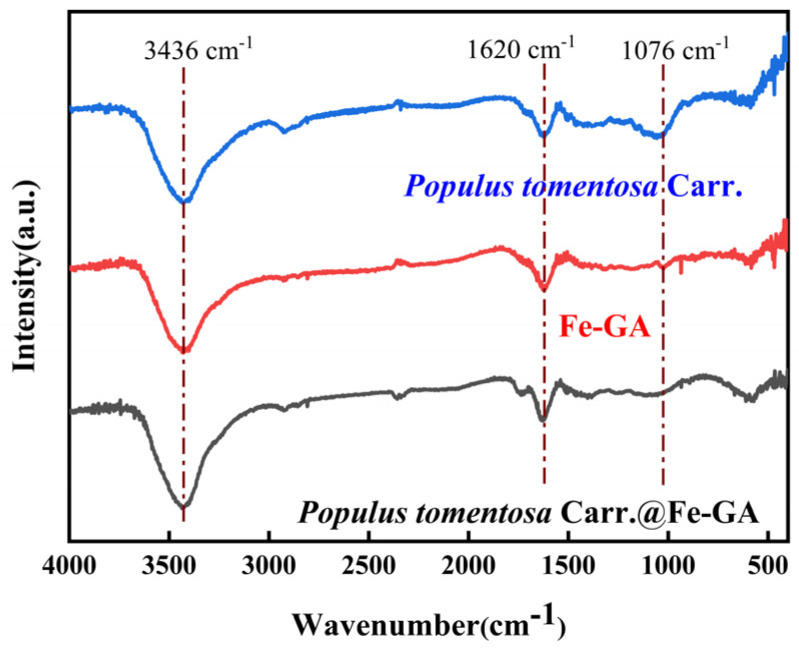
FT-IR spectra of Fe-GA, *Populus tomentosa Carr.*, and *Populus tomentosa Carr.*@Fe-GA. (*Populus tomentosa Carr.* is abbreviated to *P*).

To reveal the mechanism of *Populus tomentosa Carr.* blackening, comparative experiments and analytical characterization were conducted. Energy spectrum analysis showed that compared to *Populus tomentosa Carr.*, the O element content in *Populus tomentosa Carr.*@Fe-GA increased (Table 1), and the characteristic peak of the benzene ring was detected in the 1620 cm^−1^ region (Figure 5), indicating that TA was successfully loaded onto *Populus tomentosa Carr.* Meanwhile, Fe is detected in *Populus tomentosa Carr.*@Fe-GA, indicating the interaction between TA and Fe^3+^ and the formation of coordination compounds.

### 3.3. Thermal Stability of Populus tomentosa Carr.@Fe-GA

**Figure 6:**
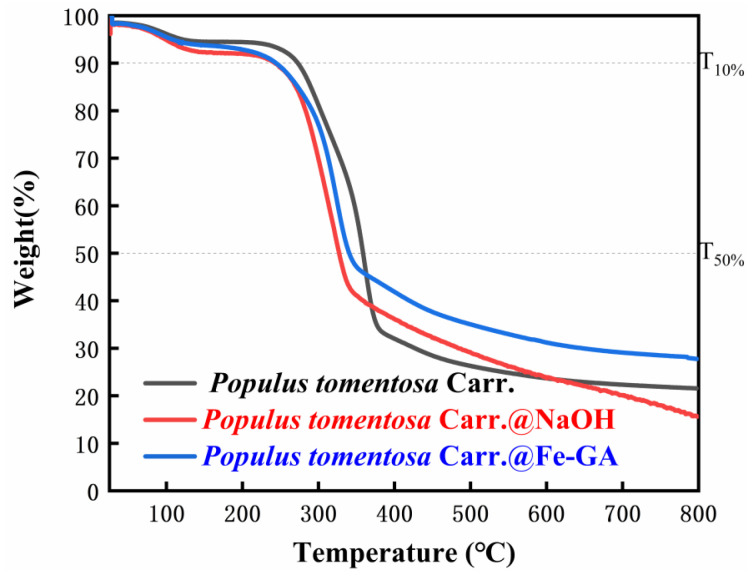
TG curves of *Populus tomentosa Carr.*, *P*@NaOH, and *P*@Fe-GA (*Populus tomentosa Carr.* is abbreviated to *P*).

The TG curves were tested under a nitrogen atmosphere (Figure 6). In the higher temperature range (30–270 °C), the curves of *Populus tomentosa Carr.* remained horizontal without significant mass changes, indicating that the wood did not degrade and possessed good thermal stability in this temperature range. Upon entering the mass loss stage, it was observed that *Populus tomentosa Carr.*@Fe-GA exhibited lower mass loss compared to *Populus tomentosa Carr.* and *Populus tomentosa Carr.*@NaOH, indicating that the prepared *Populus tomentosa Carr.*@Fe-GA was more stable than both *Populus tomentosa Carr.* and *Populus tomentosa Carr.*@NaOH, which can be attributed to the formation of tannin–iron complexes enhancing the thermal stability of the wood. Table 2 reflects the proportion of thermolysis residues of *Populus tomentosa Carr.* at high temperatures. The residual carbon rate of *Populus tomentosa Carr.* at 800 °C was 21.56%, indicating that it retained a significant amount of carbon residue at high temperatures. After NaOH treatment, the residual carbon rate significantly decreased to 15.63%, possibly due to NaOH treatment accelerating the decomposition and carbonization of the wood. In contrast, the residual carbon rate of *Populus tomentosa Carr.*@Fe-GA reached 27.77%, much higher than that of *Populus tomentosa Carr.* and *Populus tomentosa Carr.*@NaOH, further proving the significant enhancement of thermal stability due to the formation of tannin–iron complexes.

Table 3 shows that the density of *Populus tomentosa Carr.*@NaOH was lower than that of *Populus tomentosa Carr.*, indicating that NaOH successfully removed some lignin and hemicellulose. The values of *Populus tomentosa Carr.*@Fe-GA and *Populus tomentosa Carr.*@NaOH were equivalent. This indicates that iron successfully replaced the active sites of sodium ions during the impregnation process and reacted with tannic acid to form a complex uniformly loaded in the pores of *Populus tomentosa Carr.*, which is consistent with SEM data.

### 3.4. Populus tomentosa Carr.@Fe-GA Seawater Evaporation Simulation

**Figure 7:**
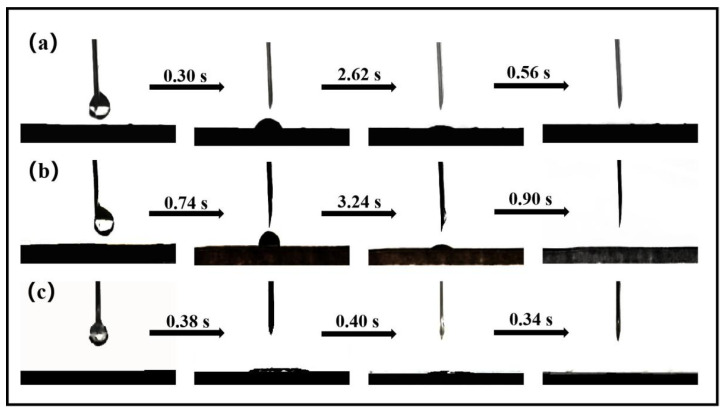
Wettability performance test of (**a**) *Populus tomentosa Carr.*; (**b**) *P*@NaOH; and (**c**) *P*@Fe-GA (*Populus tomentosa Carr.* is abbreviated to *P*).

Figure 7 compares the tested contact angles of *Populus tomontosa Carr.*, *Populus tomontosa Carr.*@NaOH, and *Populus tomontosa Carr.*@Fe-GA, revealing that *Populus tomentosa Carr.*@Fe-GA had better hydrophilicity and excellent wetting properties. The elimination of hydrophobic lignin and certain hemicellulose by NaOH may have resulted in an increased number of hydroxyl groups on the surface of the *Populus tomentosa Carr.* structure, which was made up of leftover hydrophilic cellulose fibers, thereby facilitating the deposition of TA. The formed tannin–iron complex interacted with water molecules to form hydrogen bonds, encouraging the absorption and passage of water molecules on the surface of the tannin–iron. This enabled water molecules to be swiftly moved from the bottom to the top through the transport channel that was infused with tannin–iron, giving *Populus tomontosa Carr.* specific structural characteristics and providing sufficient capillary force for seawater transport.

**Figure 8:**
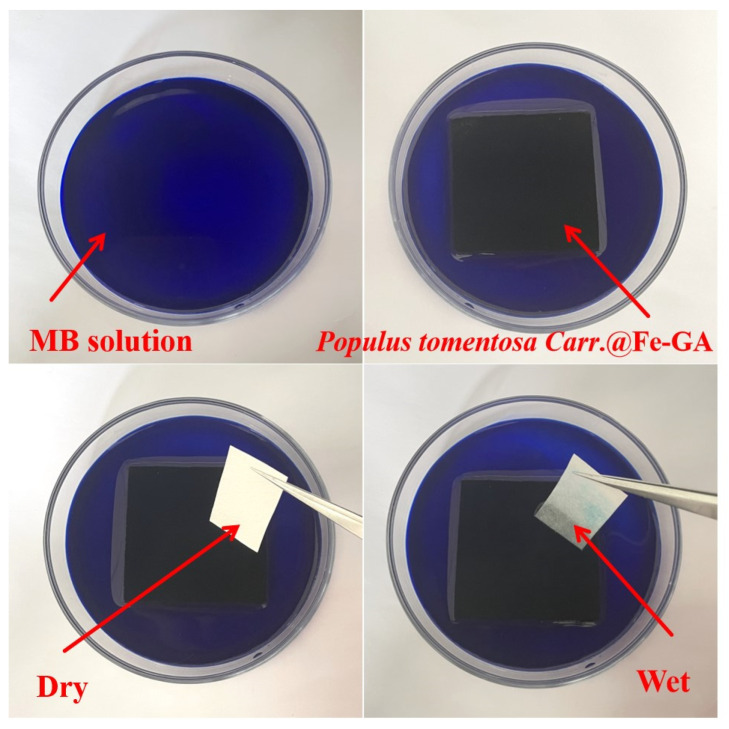
Extraction test of methylene blue (MB) solution.

As shown in Figure 8, the bottom of *Populus tomontosa Carr.*@Fe-GA was immersed in a methylene blue aqueous solution. When the absorbent paper came into contact with the upper surface of *Populus tomontosa Carr.*@Fe-GA, it could quickly extract the methylene blue solution vertically from the bottom through the water transport channel of the wood itself. At the same time, the extracted solution was mostly water-soluble, indicating that *Populus tomontosa Carr.*@Fe-GA has excellent adsorption and hydrophilicity. Its vertical water transport channel helped to transport water upward, providing sufficient water for the continuous output of wood-based photothermal materials. In addition, it also proves that *Populus tomontosa Carr.*@Fe-GA prepared in this paper had excellent firmness.

**Figure 9:**
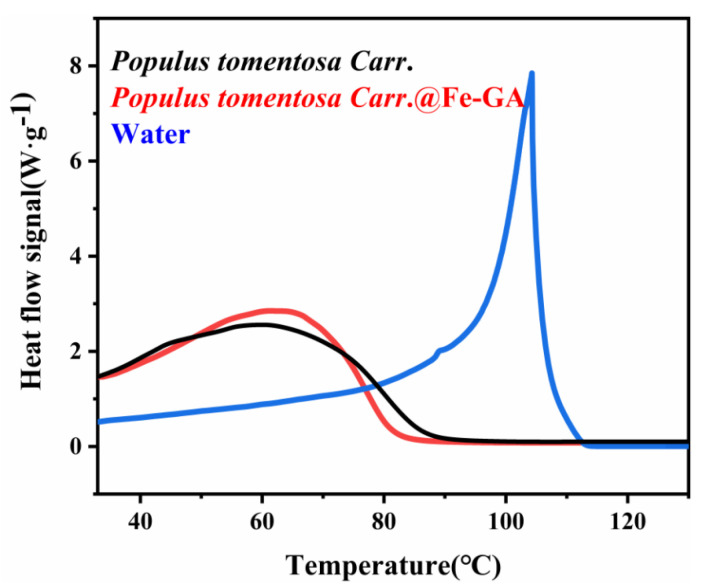
DSC curve of *Populus tomentosa Carr.*, *Populus tomentosa Carr.*@Fe-GA, and water. (*Populus tomentosa Carr.* is abbreviated to *P*).

By measuring the DSC curves of pure water, *Populus tomentosa Carr.*, and *Populus tomentosa Carr.*@Fe-GA, the evaporation behavior of the internal water molecules was studied. As shown in Figure 9, fitting the obtained curves revealed that for pure water, the heat flow signal sharply decreased after reaching its maximum value, indicating that the evaporation of water was completely finished. In contrast, the heat flow peaks of *Populus tomentosa Carr.* and *Populus tomentosa Carr.*@Fe-GA are broader, and the heat flow signal decreased slowly after reaching its maximum value, indicating that the evaporation behavior of water in the wood was completely different from that of pure water. The measured evaporation enthalpy values for the water in *Populus tomentosa Carr.* and *Populus tomentosa Carr.*@Fe-GA were 775 J/g and 748 J/g, which were significantly lower than the evaporation enthalpy value of pure water (2450 J/g). These results suggest that the large number of hydroxyl groups on the surface of *Populus tomentosa Carr.* promoted the deposition of tannic acid, which interacted with water molecules through hydrogen bonding, forming tannin–iron complexes with Fe^3+^, thereby altering the evaporation behavior of the water molecules.

**Figure 10:**
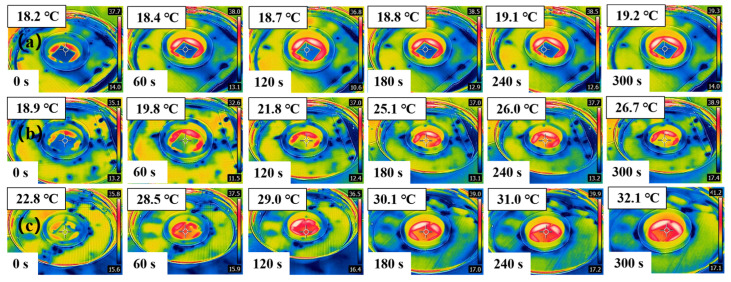
Infrared thermometer images. (**a**) Simulated seawater (3.5% NaCl). (**b**) *Populus tomentosa Carr.* (**c**) *Populus tomentosa Carr.*@Fe-GA temperature rise evolution process. (The sampling interval was established as 1 min, and the recorded solar power density was defined as one sun). (*Populus tomentosa Carr.* is abbreviated to *P*).

Figure 10a–c show that under one sun, the surface center temperature of *Populus tomentosa Carr.*@Fe-GA increased to ~32.1 °C within 300 s, while within the same time interval, the simulated seawater and *Populus tomentosa Carr.* surface center temperature could only reach~19.7 °C and 26.7 °C, respectively. *Populus tomentosa Carr.*@Fe-GA showed a high thermal response and excellent temperature rise rate, with potential photothermal evaporation characteristics.

**Figure 11:**
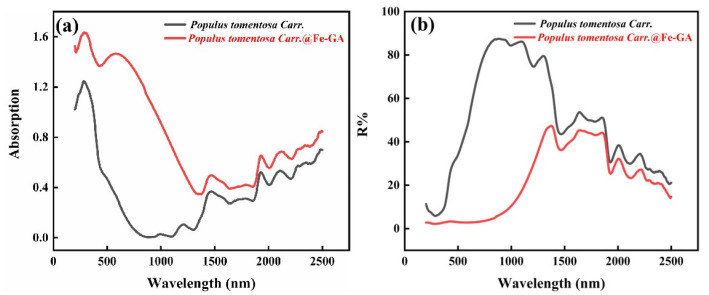
UV diffuse reflectance. (**a**) Absorption. (**b**) Reflectance spectra of *Populus tomentosa Carr.* and *Populus tomentosa Carr.*@Fe-GA. (*Populus tomentosa Carr.* is abbreviated to *P*).

As shown in Figure 11a, *Populus tomontosa Carr.* exhibited high absorption performance only in the ultraviolet region below 400 nm and reacted with TA-Fe^3+^ to form *Populus tomentosa Carr.*@Fe-GA which exhibited excellent light absorption capability in the entire solar spectrum range (200–800 nm). Within the corresponding wavelength range, *Populus tomentosa Carr.*@Fe-GA did not exhibit outstanding reflection behavior (Figure 11b), indicating that this photothermal blackening material could effectively convert absorbed solar radiation into thermal energy. By utilizing the combined effects of photothermal conversion and natural convection, evaporation was enhanced and thermal energy was conducted to seawater, increasing the water evaporation rate; this aligns with the findings presented in the water loss graph.

**Figure 12:**
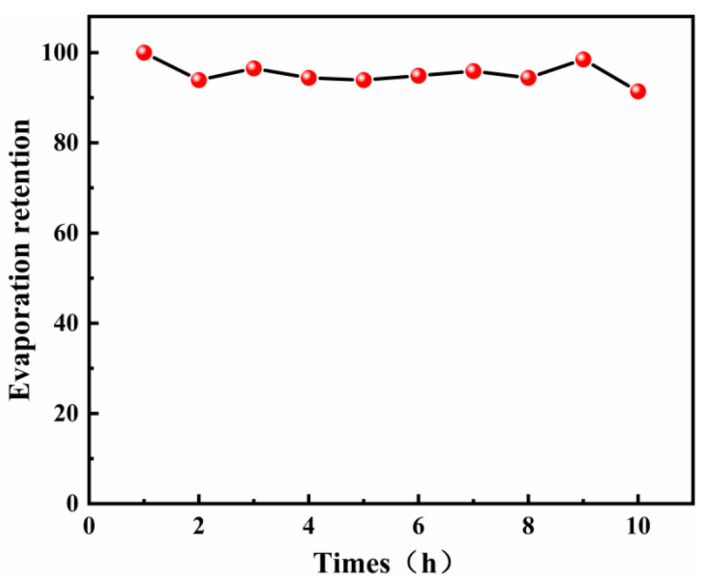
Water evaporation rate of *Populus tomentosa Carr.*@Fe-GA after continuous rinsing for 720 h (after 10 cycles with 1 h interval between each cycle). (*Populus tomentosa Carr.* is abbreviated to *P*).

After 720 h of continuous rinsing, the moisture evaporation rate retention rate of *Populus tomentosa Carr.*@Fe-GA was tested over 10 cycles (Figure 12). It can be noted that the rate of moisture evaporation for *Populus tomentosa Carr.*@Fe-GA remained very stable after 720 h of rinsing, with an average moisture evaporation retention rate of 95.4%, indicating that *Populus tomentosa Carr.*@Fe-GA prepared in this study exhibited excellent sustainability.

An evaluation of the effectiveness of wood-derived photothermal materials created using different techniques (Table 4) shows that the materials developed in this study still possessed excellent evaporation efficiency (95.1%) in a 3.5% NaCl solution, and the moisture evaporation rate was also exceptional in the area of wood-based photothermal materials. This work employed a simple and environmentally friendly method to uniformly load the complex formed by the reaction of iron elements with tannic acid onto the Populus tomentosa matrix. This photothermal blackening material exhibited excellent light absorption capabilities, efficiently transforming captured solar energy into heat, which was subsequently transferred to seawater, facilitating synergistic evaporation through photothermal conversion and natural convection, thus achieving seawater desalination [52,53]. This research provides a new solution to the global freshwater shortage issue and injects new vitality into the development of photothermal conversion technology and other renewable energy technologies. Future research can continue to optimize the pore structure of the wood to further enhance performance.

## 4. Conclusions

This work presents an effective and straightforward method for creating wood-based photothermal material. By using tannic acid and Fe^3+^, *Populus tomentosa Carr.* can be transformed into photothermal blackening material at room temperature, attributed to the formation of tannin–iron complexes (TA-Fe^3+^ coordination compounds). Experimental results show that the prepared *Populus tomentosa Carr.*@Fe-GA achieved a moisture evaporation rate of 1.72 kg·m^−2^·h^−1^ in a 3.5% NaCl solution under conditions of one sun, an increase of 44.5% compared to untreated *Populus tomentosa*. It could reach a photothermal conversion efficiency of 95.1%, which was a 53.6% enhancement compared to untreated *Populus tomentosa Carr.*, and maintain stability and high evaporation performance (95.4%) even after prolonged rinsing. This not only guarantees effective and durable solar evaporation capabilities but also offers new material options for seawater desalination technology, opening up new avenues for the functional utilization of natural resources such as wood. This work highlights the ecological sustainability and excellent thermal stability of wood, significantly enhancing its practicality and environmental applicability, achieving the functional and sustainable utilization of wood. This work offers innovative methods for creating and utilizing wood-based photothermal material, which could help address freshwater scarcity. Future research may further expand the application of this material in wastewater treatment and air purification fields.

## Figures and Tables

**Table 1:** Chemical composition of *Populus tomentosa Carr.* and *Populus tomentosa Carr.*@Fe-GA. (*Populus tomentosa Carr.* is abbreviated to *P*).

Materials	Composition (at%)		
	C	O	Fe
*Populus tomentosa Carr.*	61.0	39.0	/
*Populus tomentosa Carr.*@Fe-GA	54.1	44.2	1.7

**Table 2:** Carbon residue rates of *Populus tomentosa Carr.*, *P*@NaOH, and *P*@Fe-GA at T_10%_, T_50%_, and 800 °C (*Populus tomentosa Carr.* is abbreviated to *P*).

Materials	T_10%_ (°C)	T_50%_ (°C)	800 °C Residual Carbon Rate (%)
*Populus tomentosa Carr.*	271.7	358.7	21.56
*P*@NaOH	242.7	327.1	15.63
*P*@Fe-GA	244.2	340.6	27.77

**Table 3:** Density table of *Populus tomentosa Carr.*, *P*@NaOH, and *P*@Fe-GA (*Populus tomentosa Carr.* is abbreviated to *P*).

Materials	*Populus tomentosa Carr.*	*P*@NaOH	*P*@Fe-GA
Density (g/cm^3^)	0.56	0.458	0.454

**Table 4:** A comparison of the performance of *Populus tomentosa Carr.*@Fe-GA and other wood-based photothermal materials created using different methods in this research. (*Populus tomentosa Carr.* is abbreviated to *P*).

Light Intensity	Sample	Reference	Weight Change Rate (kg·m^−2^·h^−1^)	Evaporation Efficiency (%)
Under one sun (1 kW·m^−2^)	A facile, self-floating, lignin-based, carbon, Janus evaporator	[22]	1.539	95.88
	A lignin-/wood-based solar evaporator (LWE)	[29]	1.93	91.74
	A solar interfacial evaporator with a Janus structure	[34]	1.3	80
	A microsized, natural, wood channels solar evaporator,	[48]	1.08	75
	*Populus tomentosa Carr.*@Fe-GA	This work	1.72	95.1

## Data Availability

The original contributions presented in this study are included in the article. Further inquiries can be directed to the corresponding authors.
